# Instantaneous Material Classification Using a Polarization-Diverse RMCW LIDAR

**DOI:** 10.3390/s24175761

**Published:** 2024-09-04

**Authors:** Cibby Pulikkaseril, Duncan Ross, Alexander Tofini, Yannick K. Lize, Federico Collarte

**Affiliations:** Baraja Pty Ltd., Suite 303, Building 1, 3 Richardson Pl., North Ryde, NSW 2113, Australia

**Keywords:** LIDAR, laser sensing, polarization, classification, material properties

## Abstract

Light detection and ranging (LIDAR) sensors using a polarization-diverse receiver are able to capture polarimetric information about the target under measurement. We demonstrate this capability using a silicon photonic receiver architecture that enables this on a shot-by-shot basis, enabling polarization analysis nearly instantaneously in the point cloud, and then use this data to train a material classification neural network. Using this classifier, we show an accuracy of 85.4% for classifying plastic, wood, concrete, and coated aluminum.

## 1. Introduction

Light detection and ranging (LIDAR) is a critical sensor used by autonomous vehicles, as it provides a dense pointcloud with exceptional angular resolution, enabling the ability to provide mapping as well as detection and classification of moving objects in the environment [1]. Each point in the pointcloud is a detection event, where the LIDAR sensor has emitted energy, and received some portion of the reflected energy, using the time delay between these two events to calculate an accurate estimate of distance.

Next-generation LIDAR will use homodyne or coherent detection in the receiver hardware; this approach has several advantages over direct detection systems, such as the ability to instantaneously measure the Doppler velocity of moving targets [1]. This is possible as a homodyne detection system is able to measure the amplitude and phase of the reflected light, which provides the LIDAR sensor with additional information about the objects in the environment. In contrast, direct detection systems are sensitive to the intensity of the received signal, which is sufficient for ranging, but cannot measure the Doppler shift from a moving object.

Autonomous vehicles use sensors to understand the world around them, and in many applications, understanding the physical properties of the environment can greatly improve their functionality, such as when a sensor can classify a detection by the material type or structure; we call this ability “material classification”. This capability has been demonstrated in several autonomous applications, such as using feedback from force sensors in robotic excavators [2], using robotic arms and optical sensors in recycling plants [3], or capturing infra-red (IR) spectra of biomass on a production line to understand the composition of the feedstock [4]. Other methods of active sensing for material classification have been demonstrated with thermal sensors [5] as well as millimeter-wave vibrometry [6].

Material classification using laser sensors has shown tremendous potential; compared with camera-based methods, which are lighting dependent and rely on visible color [7], lasers provide a stimulus to the material, and then the sensor receiver records the response. Typically, the reflection from an objects is treated as an ideal Lambertian surface, which is a diffuse reflector, but real-world objects have complicated behaviour that can be characterized and used to identify materials [8]. Kirchner et al. demonstrated the ability to classify five materials using the depth error over angle and intensity from a commercial laser rangefinder [9]. Similarly, intensity histograms have been used in aerial LIDAR to classify different types of forest, as well as surfaces, such as water, gravel, and low vegetation, using a simple decision tree classifier [10].

Several authors have looked at using off-the-shelf time-of-flight (ToF) cameras to exploit depth errors for classifying materials in an image, independent of the material color [7,11]. Tanaka et al. also demonstrated that the accuracy could be greatly improved from 55.0% to 89.9% by sweeping the modulation frequency as well [7].

The use of spectral methods to classify materials is well covered in the literature and is demonstrated in diverse methods, such as hyperspectral cameras for material identification [12,13], optical absorbance sensors for detecting heavy metals in water [7], and many others. For the purpose of this article, we will focus on single wavelength LIDAR systems, as this reduces the complexity and cost of the system, instead of requiring an array of lasers or swept-wavelength systems.

Polarization is an additional property of light that describes the orientation of the oscillation of an electromagnetic wave; when reflected back from an object, the polarization state may change in a manner that is related to the physical structure of the surface of that object [14]. This insight led to investigations into how to leverage polarization LIDAR to measure the depolarization of returns from different particles. Simply stated, Mie scattering from spherical particles results in the reflected light maintaining the same polarization as the transmit beam; when the particles are non-spherical, some proportion of the reflected light is depolarized [14]. In a specific example, Sassen et al. demonstrated using polarization LIDAR to measure the ash size distribution from a volcanic eruption off the coast of Alaska [15]. Alternatively, simply augmenting LIDAR with a passive polarimetric sensor was shown to provide over 90% accuracy in classifying materials, even in low signal-to-noise (SNR) conditions [16]; in this article, the authors demonstrate the large improvement in classification accuracy by combining polarization with the LIDAR information.

Using polarization-coded LIDAR, Nunes-Pereira et al. demonstrated that polarization could be effectively used for the classification of common materials observed in operational domains for autonomous vehicles. To understand the effect, they conducted extensive examinations of the polarization-dependent reflectance of materials, then used optical coherence tomography (OCT) to determine the material cross-section of automotive car paints [17]. In order to reconstruct the degree of polarization, the authors used a pulsed ToF LIDAR and placed a linear polarizer in front of the optics. To capture the orthogonal polarization, they rotated the polarizer and repeated the capture, synthesizing a material-coded pointcloud by processing both polarizations.

In this article, we demonstrate a method of classifying materials using a polarization-diverse LIDAR with random modulated continuous wave (RMCW) ranging. This method enables material classification on an instantaneous shot-by-shot basis, only using the data acquired by the LIDAR sensor during the acquisition time. For the purpose of this article, we demonstrate the technique to classify a material, and describe a specific implementation using an integrated photonic chip to produce received signals for ranging and for calculating the polarization parameters required by our machine learning model; however, this method can be applied to LIDAR systems for various remote sensing applications. To the best of our knowledge, this is the first demonstration of a classification method using polarization-diverse RMCW LIDAR system that can perform instantaneous material classification.

## 2. Theory

### 2.1. Random Modulated Continuous Wave (RMCW) Ranging

RMCW ranging is a technique that avoids using narrow, high peak power pulses by spreading the same energy into a low peak power series of pulses, coded by a pseudorandom sequence, and was first described by Takeuchi et al. in 1983 [18]. When the received signal is digitized, it is simply correlated with the reference sequence, resulting in a correlation peak corresponding to the delay of the signal, which can be used to calculated the distance to target.

The polarization-diverse homodyne receiver is a much more complex system than the direct-detection scheme shown by Takeuchi et al., as we have four differential signals to digitize and combine, corresponding to X- and Y-polarizations, as well as the in-phase (I) and quadrature (Q) components. A thorough discussion of these devices and how polarization is recovered is shown by Roudas et al. [19].

Additionally, recovering our RMCW signal in a homodyne receiver is challenging due to the phase fluctuations of the laser source—while this can be ameliorated by using a narrow-linewidth laser, it is useful to have a system that is insensitive to laser linewidth, as this increases the types of lasers available for RMCW ranging. We provide a detailed discussion of detecting homodyne RMCW LIDAR signals in [20].

**Figure 1:**
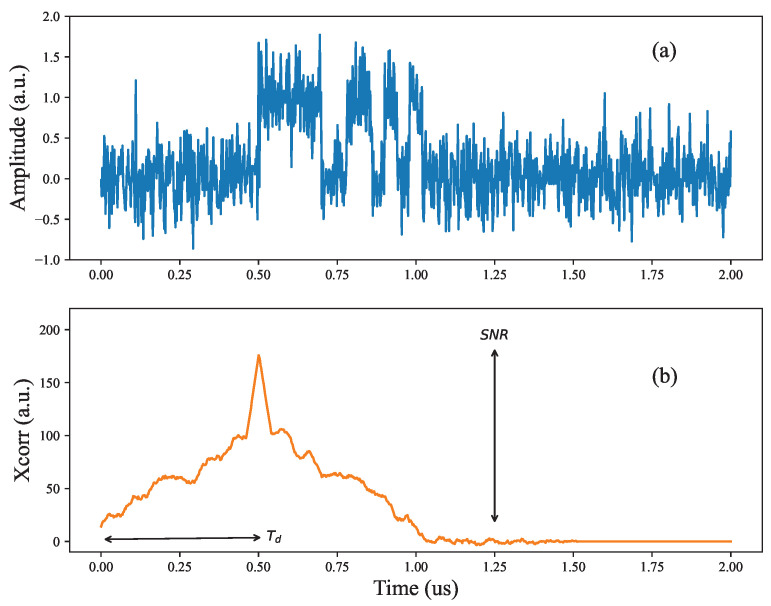
(**a**) Example of a received RMCW signal corrupted by white noise, and (**b**) the resulting correlation signal showing a peak at the delayed time of the received waveform.

As an example, we show a numerically generated example of an RMCW time domain signal converted to the correlation domain; we generated a Barker-13 code and delayed it by 0.5 μs in an acquisition window of 2.0 μs. We included additive gaussian white noise (AWGN) to introduce noise to the received signal, as shown in Figure 1a. After correlating with the ideal reference Barker-13 sequence, we obtain a correlation signal in Figure 1b, where the correlation peak corresponds to the time delay from the return signal, $T_{d}$. The distance to the target is then simply

(1) $$d_{T}=\frac{T_{d}\;\cdot \;c}{2},$$

where *c* is the speed of light. SNR is calculated as

(2) $$SNR\left(dB\right)=10\log_{10}\frac{X_{P}}{3\sigma },$$

where $X_{P}$ is the correlation peak height and σ is the standard deviation of the noise fluctuations in the correlation domain. As shown in Figure 1b, the correlation peak has quite a lot of structure outside of the main peak; this is due to the length of the Barker sequence relative to the overall acquisition time. Thus, to calculate the noise variance in σ it is important to exclude any samples that have residual correlation energy in them—for example, we use the last 200 samples to calculate σ.

### 2.2. Stokes Parameters

The in-phase and quadrature voltage signals are proportional to the electric field vector in our received optical signal, and we can, therefore, treat this as a Jones Vector $j\equiv (j_{x},j_{y})$ denoting the polarization of the transverse electric field. The zero phase of the received j will be neglected as we do not have the means to reliably measure a phase difference between transmitted and received light in a way that can isolate the dominant contributions from macroscopic propagation. This is the level of information described by Stokes Parameters [21], a four-element basis for defining polarization states in way that can be measured from optical intensity alone: $S_{0}$ as the intensity of the field, and $S_{1},S_{2},S_{3}$ as the difference in intensities of the field projected onto different common polarization bases, linear polarizations $0,90^{\circ }$ then $\pm 45^{\circ }$ and left- and right-circular polarizations.

(3) $$\begin{array}{cc} S_{0} & \equiv \bar{{\left|j\right|}^{2}}\\ S_{1} & \equiv \bar{{\left|j\;\cdot \;\left(1,0\right)\right|}^{2}}-\bar{{\left|j\;\cdot \;\left(0,1\right)\right|}^{2}}\\ S_{2} & \equiv \bar{|j\;\cdot \;\left(1,1\right)/\sqrt{2}{|}^{2}}-\bar{|j\;\cdot \;\left(1,-1\right)/\sqrt{2}{|}^{2}}\\ S_{3} & \equiv \bar{|j\;\cdot \;\left(1,i\right)/\sqrt{2}{|}^{2}}-\bar{|j\;\cdot \;\left(1,-i\right)/\sqrt{2}{|}^{2}} \end{array}$$

Here, the overline indicates the averaging over a measurement interval, which permits a fraction of power that is depolarized and not contained in $S_{1},S_{2},S_{3}$:

(4) $$\begin{array}{c} p\equiv \frac{\sqrt{S_{1}^{2}+S_{2}^{2}+S_{3}^{2}}}{S_{0}}\neq 1 \end{array}$$

In an RMCW LIDAR system, polarization variation is negligible relative to a sufficiently high sampling rate, however, the polarization variation over a codeword represents a temporal depolarization. The overline in (3) indicates averaging over a codeword.

Additionally, we assume that the launch polarization state is constant over the propagation to the target, which is a reasonable assumption given that the range to target was no more than 10 meters. In the case that this work extends to long range operation, the atmospheric effects on the launch polarization must be considered.

### 2.3. Classification Strategy

Material classification using LIDAR and polarimetric sensing has been demonstrated with a variety of classifiers, such as SVM, decision trees, and neural networks [16], showing good results in accuracy. In this work, the best performing method was SVM (accuracy = 94.4%), compared with k-nearest neighbors (accuracy = 92.0%), neural network (accuracy = 89.0%), and decision tree (accuracy = 83.8%). From this, we conclude that gains from selecting the optimal framework and training strategy are not the focus of this paper; we are investigating the applicability of this method to real-world materials and configurations that would be observed by autonomous vehicles.

Instead, we select a simple feed-forward perceptron model trained and validated using the predictive modelling tools in JMP 16 [22]. Once we have collected an entire dataset, we use a k-fold cross-validation method, where the number of folds is 5. Thus, the neural network is trained on one portion of the dataset, and then validated on the portion that has not been used for training.

As we will show in the Results section, increasing the number of nodes in the hidden layer can improve the classification accuracy; however, we would like to assess the relative performance of this classifier against different sets of materials. With this in mind, we fix our classifier to a feed-forward neural network with a single hidden layer using 64 nodes, and then try to describe the results in terms of the relative performance.

The presented neural network comprises a perceptron featuring a non-binary output classification. A total of six distinct input nodes are employed in conjunction with 64 hidden nodes. The input nodes consist of the calculated distance to the target as in (1), the SNR of the correlation peak as in (2), and the four Stokes parameters calculated from the polarization-diverse receiver, as shown in (3). A hyperbolic tangent activation function is used to facilitate the required non-binary classification. The activation function categorizes materials into output value ranges within the possible −1 to 1 overall output, depending on the number of materials for classification.

The computation of the perceptron output node’s value involves summing the inputs from the hidden nodes, each multiplied by its corresponding synaptic weight. The values of the hidden nodes are similarly calculated by summing the values of the input nodes multiplied by their respective synaptic weights, as shown in [23]. This process is handled via an automated optimizer during the training process and is documented on the JMP website [22].

## 3. Bi-Directional Optical Sub-Assembly (BOSA)

The recovering polarization from the received LIDAR signals can be accomplished with several methods [14,17,24]; however, in this work, we use a polarization-diverse homodyne receiver, similar to the work in the digital self-homodyne receiver shown by Puttnam et al. [25], but combining transmit circuit on the same chip as the receive circuit, which we call a bi-directional optical sub-assembly (BOSA). The purpose of the BOSA is to generate transmition signals in an optical fiber, and then to receive the reflected light in an optical fiber. The actual collimation into free space towards the target requires an optical telescope as well as an optical circulator to separate the transmit and receive optical signals in a coaxial LIDAR; this is detailed in Section 4.

Within the LiDAR engine, the BOSA is comprised of two primary elements: a receiver (RX) and a transmitter (TX). The transmitter segment encompasses a photonic integrated circuit (PIC) that produces a local oscillator (LO) signal and an RMCW modulated signal to be sent into the environment. Conversely, the receiver segment incorporates a photonic-integrated polarization-diverse in-phase and quadrature (IQ) receiver. Both of these elements are integrated within a PIC, ensuring a compact and efficient design.

### 3.1. Transmit Circuit (TX)

**Figure 2:**
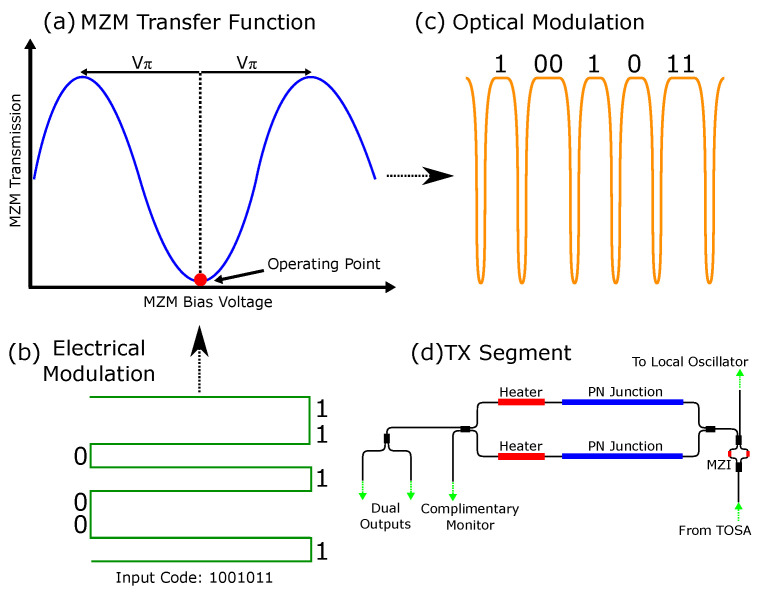
(**a**) Mach–Zehnder modulator (MZM) transfer function; operating point shown in red denotes the desired bias point to operate in phase-modulation (Vπ is the half-wave voltage). (**b**) Input electrical modulation in the form of high and low voltages. (**c**) Output optical modulation in the form of intensity and phase information. (**d**) The transmit (TX) portion of the photonic chip receives its input light from an external laser, which is then distributed between the MZM and the path leading to the local oscillator using a Mach–Zehnder interferometer (MZI).

The Tx circuit is shown schematically in Figure 2d; an external butterfly laser source is called a transmit optical subassembly (TOSA), and is coupled to the photonic chip, and then split into local oscillator and signal paths. The local oscillator is used in the receive circuitry for homodyne detection, and the signal path will be encoded with our RMCW modulation before being emitted into the environment. We use a heater-controlled Mach–Zehnder interferometer (MZI) to provide a tunable split between the two paths.

The Mach–Zehnder modulator (MZM) plays a pivotal role in transforming the electrical modulation code into optical modulation. This transformation is accomplished by modulating the depletion regions in the PN junctions of the MZM arms, as depicted in Figure 2. To enhance efficiency, a push–pull configuration is employed, effectively doubling the applied drive voltage. This method enables the attainment of the essential 2·Vπ voltage swing for phase modulation, ensuring it is achieved with the least possible power consumption. To ensure that the MZM consistently operates at the required operating point, as depicted in Figure 2a, thermal heaters are integrated into each arm of the MZM. These heaters enable fine-tuning of the MZM output, thereby maintaining continuous and stable phase modulation.

### 3.2. Receive Circuit (RX)

**Figure 3:**
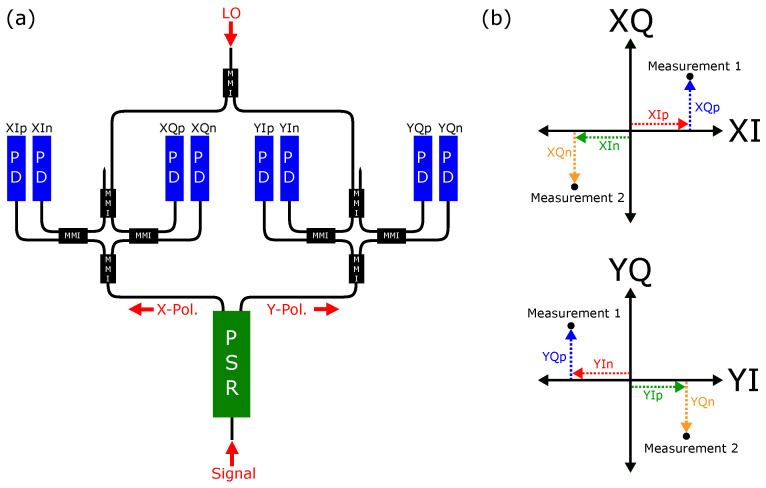
(**a**) Single channel polarization-diverse in-phase/quadrature (IQ) receiver (XIp, XIn = x-polarization in-phase pair; YIp, YIn = y-polarization in-phase pair; XQp, XQn = x-polarization quadrature pair; YQp, YQn = y-polarization quadrature pair; PSR = polarization splitter/rotator; PD = photodiode; LO = local oscillator). (**b**) X/Y polarization constellation diagrams. Two example measurements are shown with their component breakdowns, which are labelled on the receiver.

Our ability to detect and classify materials is based on accurate measurement of the polarization state of the received signal, and this Rx circuit in the BOSA is the core component that achieves this functionality. This advanced receiver comprises three principal components: a polarization–splitter–rotator (PSR), $90^{\circ }$ hybrids, and photodetectors (PDs), all interconnected via low-loss SiN waveguides within the PIC, as illustrated in Figure 3a.

PSR plays a pivotal role in processing optical inputs with indeterminate TE/TM polarization. It adiabatically transforms the TM component into the fundamental TE mode of the SiN waveguides, while simultaneously transmitting the TE component without alteration. The unaltered TE fraction is hereby referred to as the X-polarization and the adiabatically converted TM fraction is referred to as the Y-polarization. Subsequent to polarization separation, each polarization state is directed to its corresponding 90° hybrid, which consist of four multi-mode interference couplers (MMIs). A 2 × 2 MMI with an unused input port is employed on the LO side of the hybrids, whereas the signal input is managed by a 1 × 2 MMI on the opposite side.

This configuration results in a 90° phase difference between the two inputs, labelled in the diagram as *p* and *n*, essential for the receiver’s capability to process both amplitude and phase information. To convert this optical information into processable electrical information, pairs of vertically stacked germanium photodiodes are implemented, with their photocurrents subtracted to remove any common-mode noise, as shown by Jin et al. [26]. The resulting photocurrents generated across the photodiodes are then converted to voltage and amplified through the use of trans-impedance amplifiers (TIAs). Figure 3b depicts how the *p* and *n* photocurrents are $90^{\circ }$ out of phase, but construct an in-phase and quadrature measurement, which is also the case for the Y-polarization. Thus, eight photocurrents result in the digitization of four voltages at the ADC.

## 4. Experimental Setup and Method

**Figure 4:**
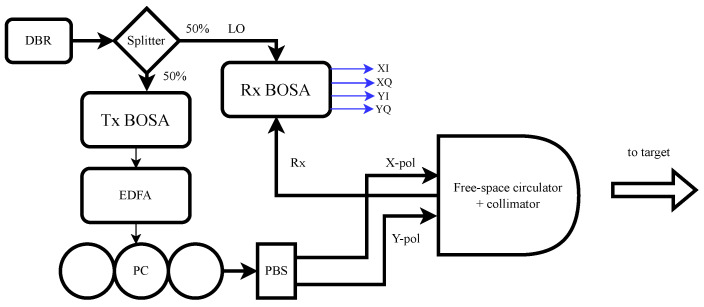
Experimental setup: we use a Tx BOSA for modulating the RMCW code on the transmit path, and an RX BOSA for performing the polarization-diverse IQ demodulation, using unmodulated light as the local oscillator (DBR: distributed Bragg reflector, EDFA: erbium-doped fiber amplifier, PC: polarization controller, PBS: polarization beamsplitter, Rx: received optical path, XI, XQ, YI, YQ: the in-phase and quadrature portions of the x- and y-polarization.).

Figure 4 displays the experimental setup used to validate our material classification approach using a single-point RMCW LIDAR system. In our experimental setup, we use a wavelength-tunable DBR laser (Oclaro TL3000), set to 1545.3 nm and with +12 dBm output power. The laser is connected to the input of our custom silicon photonic BOSA, as described in Section 3.1, which modulates the laser signal with a phase-modulated 512-bit Gold code, where the MZM is biased, as shown in Figure 2a. In this setup, the Gold code is 2 μs in duration, and then the acquisition system waits another 2 μs for the return signal. Thus, each pixel takes a total of 4 μs in duration. To boost the optical power, we use an erbium-doped fiber amplifier, which boosts the transmitted power to +27 dBm.

To control the polarization of the transmitted beam, we connect a fiber polarization controller to the output of the EDFA, and align the polarization to be $45^{\circ }$ linear relative to the input of the polarization beam splitter (PBS). Thus, we have equal power in both X- and Y-polarizations in separate fibers, which are assembled together into a fiber array. As part of our design of experiments, we would like to vary the transmitted polarization, and by connecting/disconnecting the inputs of the PBS, we are able to create roughly $0^{\circ }$, $45^{\circ }$, and $90^{\circ }$ linear polarizations.

The optical fiber array is used to closely position both Tx fibers and a single Rx fiber in parallel behind a birefringent and magneto-optic crystal stack, forming a system that resembles a free-space optical circulator. These types of non-reciprocal devices have been used in optics for decades to enable a polarization-independent separation of forward- and backwards-travelling waves; we direct the reader to the literature to understand more about these devices [27,28]. Creating a collimation system that projects both Tx and Rx fibers onto the same optical axis after collimation is called a coaxial LIDAR system, and has several benefits, such as ensuring alignment of Tx and Rx paths at all times. In this manner, the system resembles a free-space optical circulator with a single collimating lens that focuses on both the Tx and Rx fibers. The resulting collimated beam has a $1/e^{2}$ intensity diameter of 19.1 mm × 5.2 mm.

This beam is directed to the target of interest, and is reflected along the same path to the collimation optics. The free-space optical circulator collects the received signal and couples it to a singlemode fiber, which is edge-coupled to the Rx BOSA, as described in Section 3.2. The photonic integrated circuit mixes the local oscillator with the weak reflected LIDAR signal from the environment and creates four photocurrents, for two polarizations and two quadratures. These photocurrents are converted to voltages and then sampled by 4 × 500 MSps ADCs, producing a polarization-diverse field reconstruction of the received LIDAR signal, labelled as XI, XQ, YI, and YQ, where X and Y refer to the horizontal and vertical polarization, and I + Q are the in-phase and quadrature photocurrents.

These digitized signals are processed in two steps: First, combined into a single measurement and correlated digitally with the ideal RMCW code, producing a correlation signal, similar to Figure 1b. From this, we calculate the distance to thre target and the SNR. This signal processing chain is executed entirely in a Xilinx Ultrascale (ZU19) FPGA, which has been designed to accommodate the time budget of calculating the relevant parameters every 4 μs.

Second, we process the polarization measurements to calculate the Stokes time-series for the received signal, as in Section 2.2. We then select the mean Stokes values over the duration of the codeword and use scalar values of S0, S1, S2, and S3 as inputs to our machine learning model, which, combined with the distance SNR, provides six inputs to our neural network model. In this experiment, the neural network is processed offline using Python; however, the neural network that we use is a simple multi-layer perceptron with a single hidden layer. Thus, we believe that the calculation of the material class can be implemented in an FPGA and the calculation can be completed in the 4 μs acquisition window.

### 4.1. Data Collection Methodology

**Figure 5:**
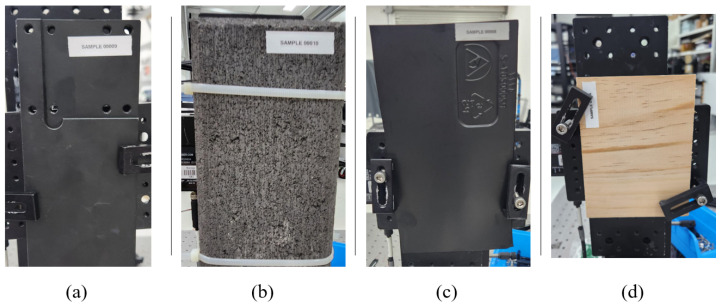
Materials used for experimental validation: (**a**) coated aluminum, (**b**) concrete, (**c**) black plastic, (**d**) engineered wood.

The set of materials used in our experiment are four that would occur in an urban environment: black power-coated aluminum, concrete, engineered wood, and black plastic; photos of the samples are shown in Figure 5. The samples are large enough for our laser beam to entirely fit without clipping. In order to assess the classification performance of the polarization-diverse RMCW LIDAR, we sought to collect data in controlled experimental conditions, but with predetermined variations that would mimic a situation in a real environment. To this end, we collected data for every material sample and varying the target distance, the angle of incidence to the LIDAR (which we call yaw), the rotation of the material in the plane perpendicular to the LIDAR optical path (which we call roll), and the polarization of the transmission signal from the RMCW LIDAR.

These deliberate variations are used to explore the range of polarizations that we receive in the BOSA, and to validate the classifier with these variations.

The variations are explored using a full-factorial design-of-experiments (DOE), and the values used in the input factors are shown in Table 1. For each one of the 108 combinations, we collect roughly 830 measurements for a total of more than 89,640 measurements per material; using our RMCW reference code, we correlate the return signal and use a constant false alarm rate (CFAR) algorithm to determine if we have a valid peak, using a false alarm rate of 1 × $10^{-4}$ [29]. If no peaks are passed through CFAR, we reject the measurement completely.

## 5. Results

As described in Section 4.1, each material was tested according to the DOE plan, with the polarization-diverse quadrature signals digitized and recorded for every configuration for each material. Thus, we stored more than 89,000 measurements for each material, and each measurement was processed to produce a distance to the target, the SNR of the cross-correlation peak, and the four Stokes parameters. Therefore, in total, we collected 356,000 measurements to train and validate our classifier, with the DOE ensuring we had significant real-world variation in the dataset.

**Figure 6:**
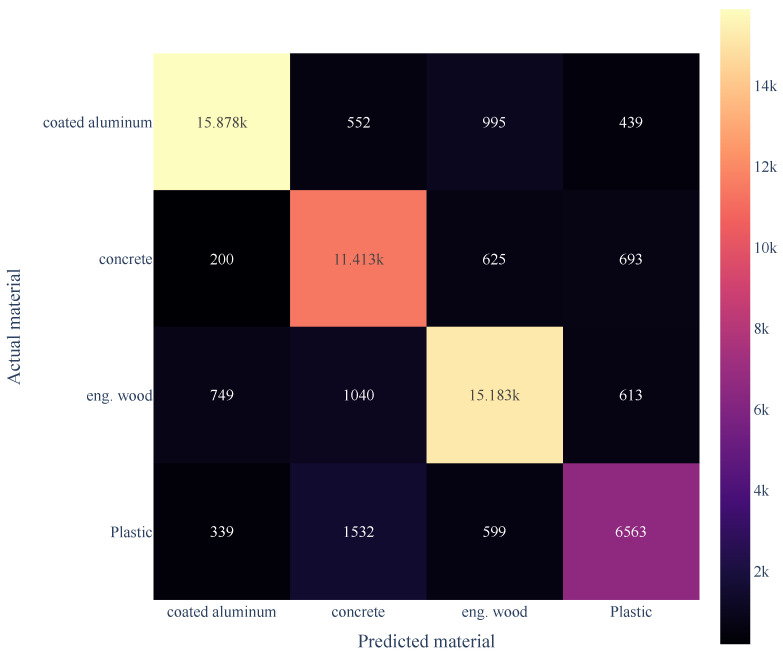
Confusion matrix for the 64-node classifier on four different materials.

These six measurement outputs are the six inputs of our neural network. We then trained a multi-layer perceptron neural network with a single hidden layer of 64 nodes, using a tanh activation function, as described in Section 2.3, and then ran the resulting model on the validation data set. We expressed the performance of the classifier with a confusion matrix, as shown in Figure 6. The overall classification accuracy was 85.4%; however, from the confusion matrix, it is clear that the majority of the misclassifications came from plastic, which had a classification accuracy of just 72.6%.

**Figure 7:**
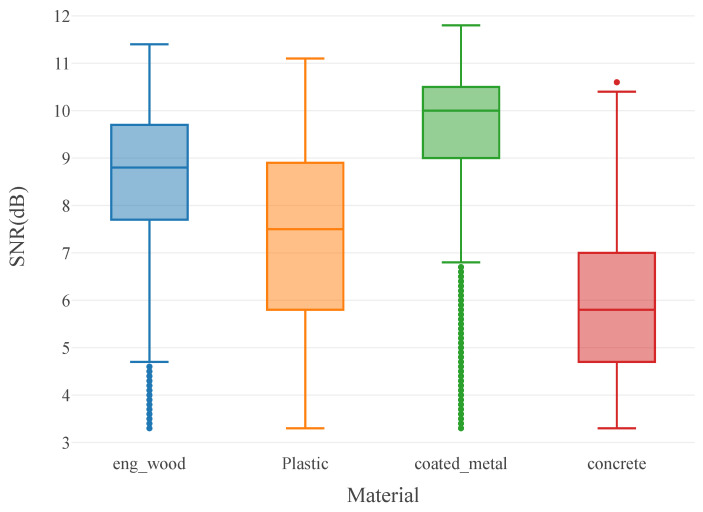
Distribution of SNR values for all materials in the dataset.

We believe that the cause of this lower accuracy for black plastic is not related to the characteristics of the material, but from the amount of measurement data in building the classifier. From the confusion matrix in Figure 6, we can see that the number of samples available for each of aluminum, concrete, and engineered wood (n = 17,864, 12,931, and 17,585, respectively) was much higher than the number of samples for black plastic (n = 9033), which produced a worse accuracy. This was expected as the number of available measurements from black plastic was much lower, thus the material classifier was biased against black plastic. This is due to the low reflectivity of the material, and in the future, a classifier should be trained on an equal number of points for every class. The difference in SNR for each material class is shown in Figure 7.

In comparison with previous work, Lee et al. used the estimation of surface reflectance from a time-of-flight camera to achieve an accuracy of 76.5% on seven materials [11]. Using a polarimetric multispectral LIDAR, Han et al. demonstrated 100% material classification, demonstrating no false detections by combining polarization information as well as the information from 33 lasing wavelengths between 580 nm and 900 nm [30]. This approach, while impressive, needs a large laboratory setup to create the multispectral LIDAR, and would not be amenable to an integrated LIDAR system.

Capturing the variation over the DOE is an important method to assess the real-world performance of the classifier. For example, we measured the materials at two distances, as we expected the laser return SNR to change over distance for each material. If we trained the classifier at a single distance of 10 m, the classification accuracy was 99.7%; similarly, training only at measurements of 3 m yielded a classification accuracy of 99.6%. In these two cases, the network was overfitted to the measurements of these materials at that specific distance, and thus the classifier would perform poorly at any other distance.

**Figure 8:**
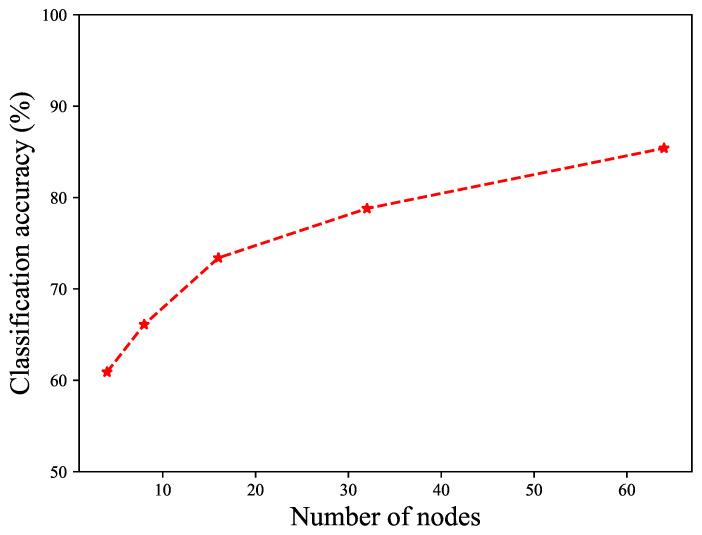
Classification accuracy as number of nodes in hidden layer increase.

To explore the performance of the classifier with the hidden node number of neurons, we repeated the training and validation for a selection of hidden layer sizes, and measured the overall classification accuracy, as shown in Figure 8.

**Figure 9:**
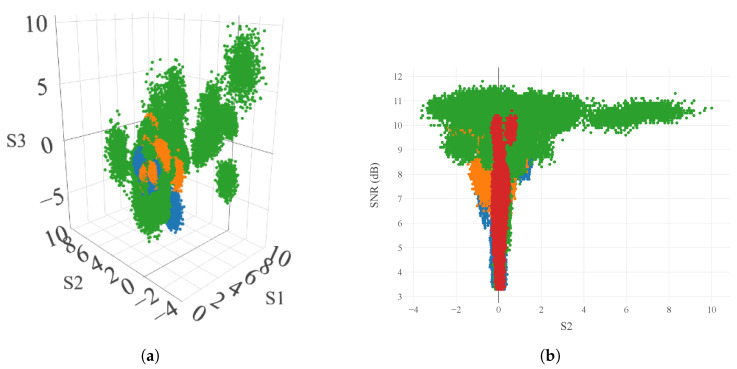
Scatter plots to demonstrate the clustering of measurements in polarization space, and with SNR. Points are colored by material, with concrete (red), coated metal (green), plastic (orange), and engineered wood (blue). (**a**) 3D visualization of S1, S2, and S3. (**b**) 2D scatter plot of SNR and S2.

We use a 3D scatter plot to visualize the Stokes parameters, S1, S2, and S3, and the measurements demonstrated that the materials showed differing polarization results; note that we remind the reader that the launch conditions were $0^{\circ }$, $45^{\circ }$, and $90^{\circ }$ linear polarization relative to the output window of the LIDAR sensor. As shown in Figure 9a, all materials measured Stokes parameters that were well clustered; the exceptions were concrete (in purple), which was clustered, but not visible, and coated metal, which was clustered into many smaller groupings. This would suggest that classification on concrete would have a poor accuracy, and that most of the false classifications would be for coated aluminum. In contrast, the confusion matrix in Figure 6 indicates a different conclusion; the accuracy for concrete was 88.3%, and the false reading was highest for plastic.

Additional insight is available by plotting the relationship between SNR and the Stokes parameters. In Figure 7, there is an observable difference in the mean SNR of each material, though the overall distributions are not separated; in Figure 9b, we see a clear distinction between concrete and the other materials, as concrete is strongly confined in S2, but greatly dispersed in SNR. Similarly, we note that coated metal only has measurement points above SNR > 8 dB.

## 6. Conclusions

We have demonstrated instantaneous material classification using an RMCW LIDAR system with an integrated transmit/receive photonic chip to enable polarization-diverse homodyne detection. This technique enables the creation of LIDAR point clouds with a point-by-point estimate of materials in the environment, which could greatly aid perception tasks for autonomous vehicles and robotics. In our field test at 3 m and 10 m, we varied the angle of incidence and rotation normal to the LIDAR, as well as the launch polarization state, collecting over 350,000 measurements to train and validate our machine learning model for material classification. The field test demonstrated that plastic, engineered wood, concrete, and coated aluminum could be correctly classified with an accuracy of 85.4%. We believe that this is a strong demonstration of material classification as a novel LIDAR data product for autonomy and robotics.

## Figures and Tables

**Table 1:** Experimental factors used in the full-factorial design-of-experiments (DOE) for collecting the training and validation data.

Input Factor	Values
Distance (m)	3, 10
Tx polarization (°)	0, 45, 90
Yaw (°)	0, 7, 15
Roll (°)	0, 45, 135, 180, 225, 315

## Data Availability

The data presented in this study are available on request from the corresponding author.
